# Harnessing the potential of radiation treatment of brain metastases to improve melanoma immunotherapy

**DOI:** 10.1186/2051-1426-3-S2-P367

**Published:** 2015-11-04

**Authors:** Corey D McNeilly, Lukas W Pfannenstiel, Chaomei Xiang, Jennifer Yu, Brian R Gastman

**Affiliations:** 1 Cleveland Clinic, Lerner Research Institute, Cleveland, OH, USA; 2 Cleveland Clinic, Cleveland, OH, USA

## Introduction

Radiation is an integral part of melanoma therapy, particularly for patients with symptomatic metastases. Efficient killing of tumor cells by radiation has been shown in immunocompromised mouse models to be enhanced greatly with transfer of a functioning immune system, particularly CD8+ T cells. In a subset of patients, addition of radiation to immunotherapy has also enhanced immune system-mediated tumor cell kill in non-irradiated areas, termed abscopal effect. This effect is thought to be a result of increased shedding of tumor antigens upon radiotherapy that primes the immune system to establish a more robust attack on melanoma cells throughout the body. Whether or not brain metastases in the blood brain barrier can be used to develop an abscopal effect is unknown. Pre-clinical reports support synergism between PD-1 inhibition and radiation in tumor killing and prolonged survival.

## Methods

To assess the extent of abscopal effect in mice models simulating brain metastasis, we measured the degree to which radiation enhances anti-PD1 immunotherapy of non-irradiated tumor control. 2.0×10^3^ luciferase-expressing B16 melanoma cells were transplanted intracranially and 8.0×10^5^ cells were transplanted subcutaneously into C57BL/6 mice to establish brain and flank tumors. Anti-PD1 and IgG treatment groups received 6 Gy irradiation to the brain or flank tumor respectively. Preliminary data suggest this dose is tolerated without obvious side effects and capable of partly reducing tumor growth. Lead shielding was used to limit radiation to vital organs and the contralateral tumor. Tumor growth was measured by IVIS bioluminescence imaging.

## Results

Anti-PD1 therapy significantly affected brain tumor growth and survival. Mean brain tumor growth was significantly lower for anti-PD1 treated groups and mean survival was longer for anti-PD1 treated mice than for IgG treated mice (26.4 vs. 19.8 days, p < 0.001). Relative to mice receiving no irradiation and those receiving both IgG treatment and head tumor irradiation, flank tumor growth rate was lower for mice receiving anti-PD1 treatment and head tumor irradiation, suggesting a possible abscopal effect. Mean flank tumor growth was lowest in mice receiving a combination of anti-PD1 treatment and flank tumor irradiation. Anti-PD1 treatment was also associated with enhanced numbers of CD8+ T cells in the tumor microenvironment.

## Conclusion

This is the first study to show that melanoma brain metastases, commonly radiated for palliation, have the potential to enhance systemic immunotherapy effects on non-irradiated melanoma tumors. Ongoing studies will examine additional melanoma cell lines as well as immunological changes in the microenvironment and T cell function.

